# Supplementary material for: Biomolecular condensate phase diagrams with a combinatorial microdroplet platform
Source: Nat Commun. 2022 Dec 21;13:7845. doi: 10.1038/s41467-022-35265-7 (PMC9768726; doi:10.1038/s41467-022-35265-7)
Supplement: Supplementary file 1 — Supplementary Information [file 41467_2022_35265_MOESM1_ESM.pdf]

Supplementary Information: High Resolution and Multidimensional Protein Condensate Phase Diagrams with a Combinatorial Microdroplet Platform

William E. Arter,[1,†] Runzhang Qi,[1,†] Nadia A. Erkamp,[1,†] Georg Krainer,[1,†] Kieran Didi,[1] Timothy J. Welsh,[1] Julia Acker,[2] Jonathan Nixon-Abell,[3] Seema Qamar,[3] Jordina Guillén-Boixet,[4] Titus M. Franzmann,[4] David Kuster,[5] Anthony A. Hyman,[5] Alexander Borodavka,[2] Peter St George-Hyslop,[3,6,7] Simon Alberti[4] and Tuomas P.J. Knowles[1,8,*]

[1] Yusuf Hamied Department of Chemistry, Centre for Misfolding Diseases, University of Cambridge, Lensfield Road, Cambridge, CB2 1EW, UK

2 Department of Biochemistry, University of Cambridge, Cambridge, CB2 1QW, UK

3Cambridge Institute for Medical Research, Department of Clinical Neurosciences, University of Cambridge, Cambridge CB2 0XY, UK

[4] Biotechnology Center (BIOTEC), Center for Molecular and Cellular Bioengineering (CMCB), Technische Universität Dresden, Tatzberg 47/49, 01307 Dresden, Germany

[5]Max Planck Institute for Molecular Cell Biology and Genetics, Pfotenhauerstrasse 108, 01307 Dresden, Germany

6 Department of Medicine (Division of Neurology), University of Toronto and University Health Network, Toronto, Ontario M5S 3H2, Canada

[7]Department of Neurology, Columbia University, 630 West 168th St , New York, NY 10032, USA.

[8] Cavendish Laboratory, Department of Physics, University of Cambridge, J J Thomson Ave, Cambridge, CB3 0HE, UK

[†] These authors contributed equally.

* To whom correspondence should be addressed:
E-mail: tpjk2@cam.ac.uk

Fluorescence recovery after photobleaching of FUS^{G156E}-EGFP condensates

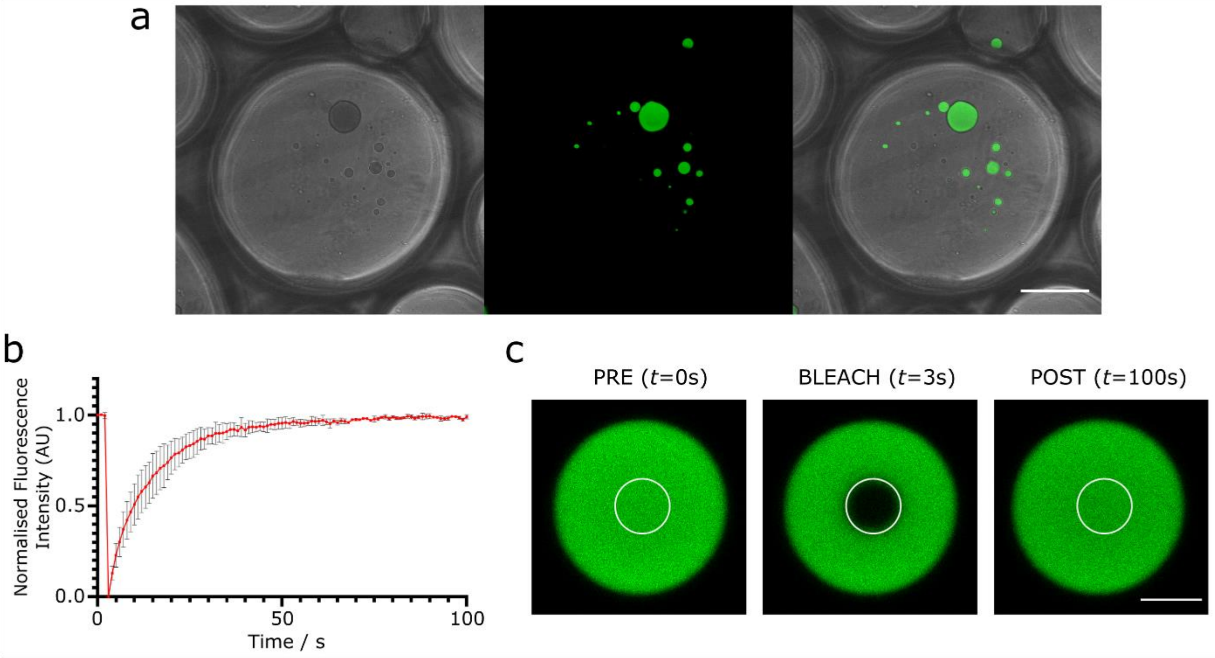

Supplementary Figure 1. Fluorescence recovery after photobleaching of FUS^{G156E}-EGFP condensates. (a) Brightfield (left), fluorescence (centre), and merged (right) fluorescence microscopy images of water-in-oil droplets containing FUS^{G156E}-EGFP condensates. Scale bar 50 µm.
(b) Fluorescence recovery data for FUS^{G156E}-EGFP condensates encapsulated in water-in-oil droplets. Data are presented as mean values ± standard deviation of three FRAP acquisitions of different condensates. Scale bar 5 µm.. **(c)** Exemplary FRAP data before (PRE, *t* = 0s), shortly after photo bleaching pulse (BLEACH, *t* = 3s), and after fluorescence recovery (POST, *t* = 100s).

Supplementary Note 1

The liquid nature of homotypic FUS condensates (4 µM FUS^{G156E}-EGFP, 4% *w/v* PEG 6k) encapsulated within oil-in-water microdroplets was confirmed by fluorescence recovery after photobleaching (FRAP) experiment (Supplementary Figure 1). Fluorescence recovery occurred within 50 ms (mobile fraction 98.3 ± 0.63 %), indicating that condensates retained their liquid nature when encapsulated within fluorinated oil in the presence of 1.5% fluorosurfactant.

Comparison between PhaseScan and manual measurements

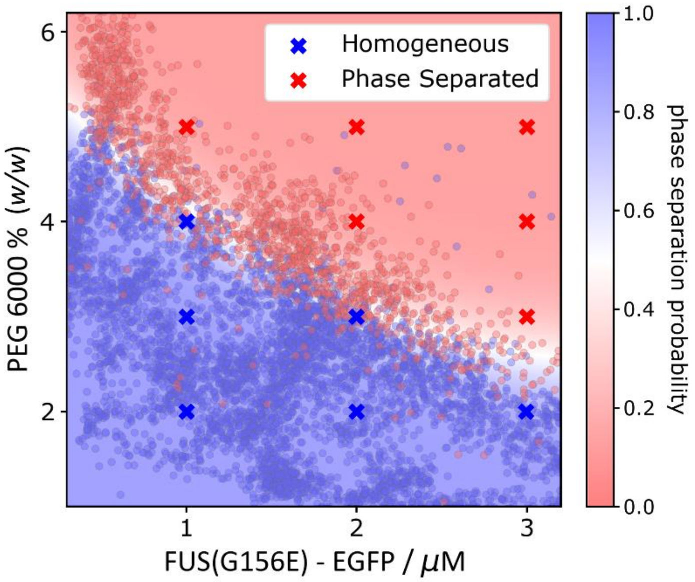

Supplementary Figure 2. Comparison between PhaseScan and manual measurements. The crowding-induced homotypic phase separation of EGFP-tagged FUS^{G156E} in the presence of PEG 6000 was analysed by independent PhaseScan (dots and colour map) and manual pipetting experiments (crosses). All points were observed to match the phase diagram as determined by PhaseScan.

Supplementary Note 2

To verify that the phase diagram generated by the PhaseScan platform is in accordance with the phase diagram obtained from bulk experiments, we performed manual pipetting experiments with FUS^{G156E} and compared the phase behaviour with the data obtained by PhaseScan (Supplementary Figure 2). We performed manual bulk measurements at twelve different points in the phase diagram, stock solutions of buffer, 6k PEG (20%) and FUS (18.23 μM as verified by Nanodrop measurements at 488 nm) were prepared and mixed to give 10 μL of each of the sample compositions as shown in Supplementary Figure 2. The sample was pipetted onto a microscope slide equipped with imaging chambers, which was sealed with a coverslip, before imaging EGFP fluorescence with a 10× objective. The images were manually classified as containing phase-separated or homogeneous protein and compared to the corresponding region in the phase diagram produced by the PhaseScan platform. All manually determined datapoints match well with the boundary as determined by PhaseScan platform.

Effect of barcoding dyes on phase behaviour

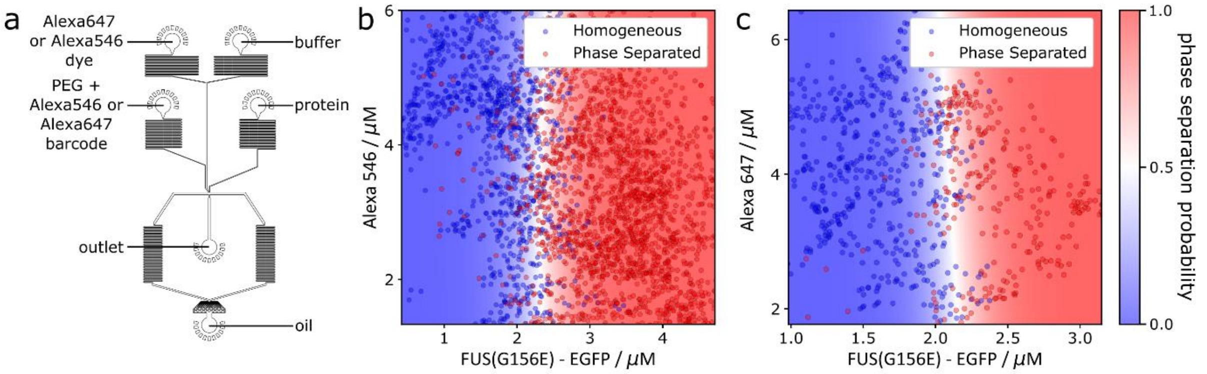

Supplementary Figure 3. Effect of barcoding dyes on phase behaviour. (a) PhaseScan chip used to assess dye behaviour. **(b)** Phase diagram of EGFP-tagged FUS^{G156E} vs. increasing concentrations of the barcoding dye Alexa 546. **(c)** Phase diagram of EGFP-tagged FUS^{G156E} vs. increasing concentrations of the barcoding dye Alexa 647. Source data is provided as a Source Data file.

Supplementary Note 3

To assess whether the fluorescent dyes used to barcode for different PhaseScan solution components effect phase behaviour, control experiments were performed in which EGFP-FUS^{G156E} protein and barcoding dye concentrations were varied in the presence of a single concentration (2.4% (*w*/*w*)) of PEG 6000. This was achieved through the use of a 4-variable droplet generator as shown in Supplementary Figure 3(a). We introduced Alexa647 or Alexa546 dye, buffer and protein solutions into the chip with varying flow rates according to an automated flow programme (total flow rate 60 μL/h) with the Alexa546 or Alexa647-barcoded PEG solution injected into the chip at a rate of 7 μL/h. Droplets were collected and analysed as described in the Methods. Data points possessing anomalous PEG barcode concentrations (due to variance in the PEG flow rate) were removed, and the resultant phase diagrams for EGFP-FUS^{G156E} concentration vs. Alexa546 or Alexa647 concentrations were produced (Supplementary Figure 3(b, c)), respectively). We observed no significant variation in the position of the phase boundary with respect to Alexa546 or Alexa647 dye concentration, indicating that the phase behaviour is independent of barcode dye concentration.

Time dependence, reproducibility, and minimal effect of outliers

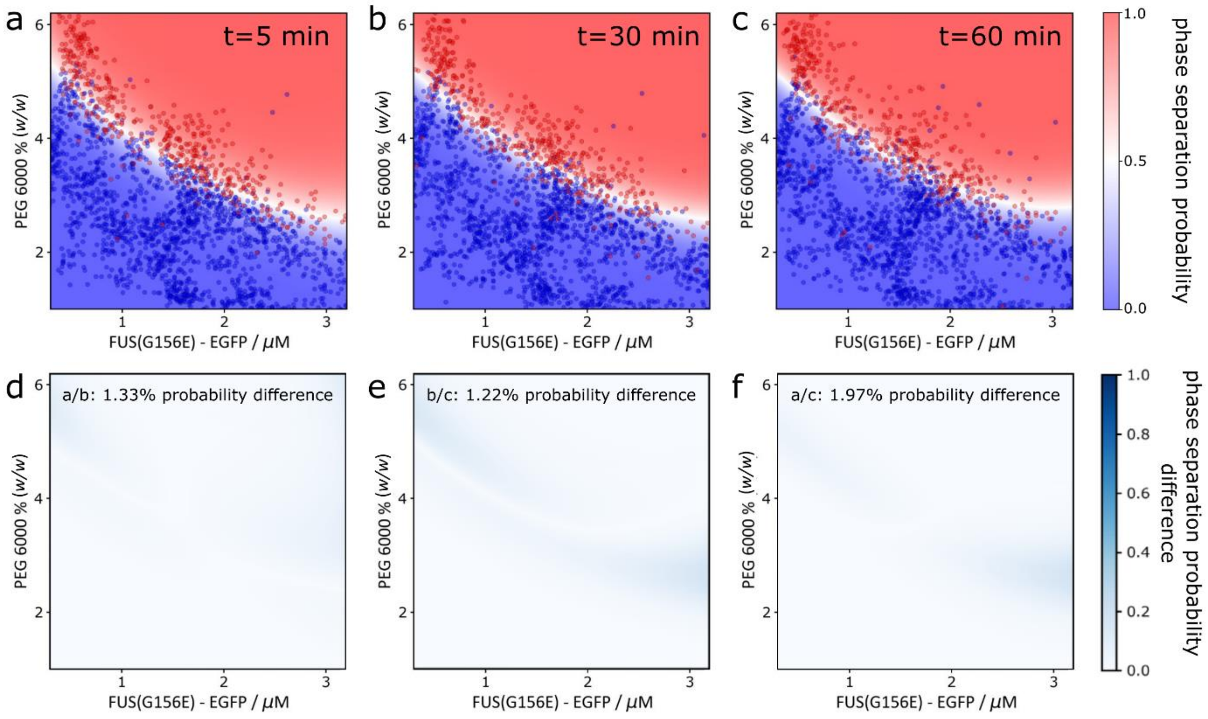

Supplementary Figure 4. Reproducibility of PhaseScan method, minimal effect of outliers, and time-dependence of phasescan measurement. (a–c) Phase diagrams corresponding to separate phase scan experiments with normalised number of droplets (N = 2754), with data acquisition after drop generation of (a) 5 min, (b) 30 min (N = 2745 droplets) and (c) 60 min. (d–f) Plots comparing reported phase separation probability in experiments panels (a)–(c), average difference 1.51 ± 0.33 % mean ± standard deviation, N = 3. Source data is provided as a Source Data file.

Supplementary Note 4

To investigate the reproducibility and time-dependency of phase diagram generation, three phase scan experiments were conducted with the same reagent inputs, before imaging data was acquired 5, 30 and 60 min after droplet generation (Supplementary Figure 4). Only negligible differences are observed in the integrated phase separation probability between the repeats (average difference 1.51 ± 0.33 % (mean ± standard deviation, N = 3)), which were randomly sampled to contain the same number of datapoints in each dataset (N = 2754). This demonstrated reproducibility of droplet production, trapping, data acquisition and analysis. Notably, although a small number of incorrectly classified or barcoded outliers exist in all three datasets, these datapoints have negligible effect on the reported position of the phase boundary since they exist as only a small (< 2%) proportion of the overall dataset.

The negligible differences between the phase diagrams shows that there is no time-dependence in the observed phase behaviour, confirming that the phase diagrams are collected under equilibrium conditions.

In addition, by randomly sub-sampling each dataset further (i.e. bootstrapping) to include N = 1500 datapoints, less than the smallest dataset presented in this manuscript (Figure 2(f), N = 1599), we found that the probability difference between sampling repeats remained < 2.75 ± 0.74 % (mean ± standard deviation, N = 5).

Effect of droplet size on phase behaviour

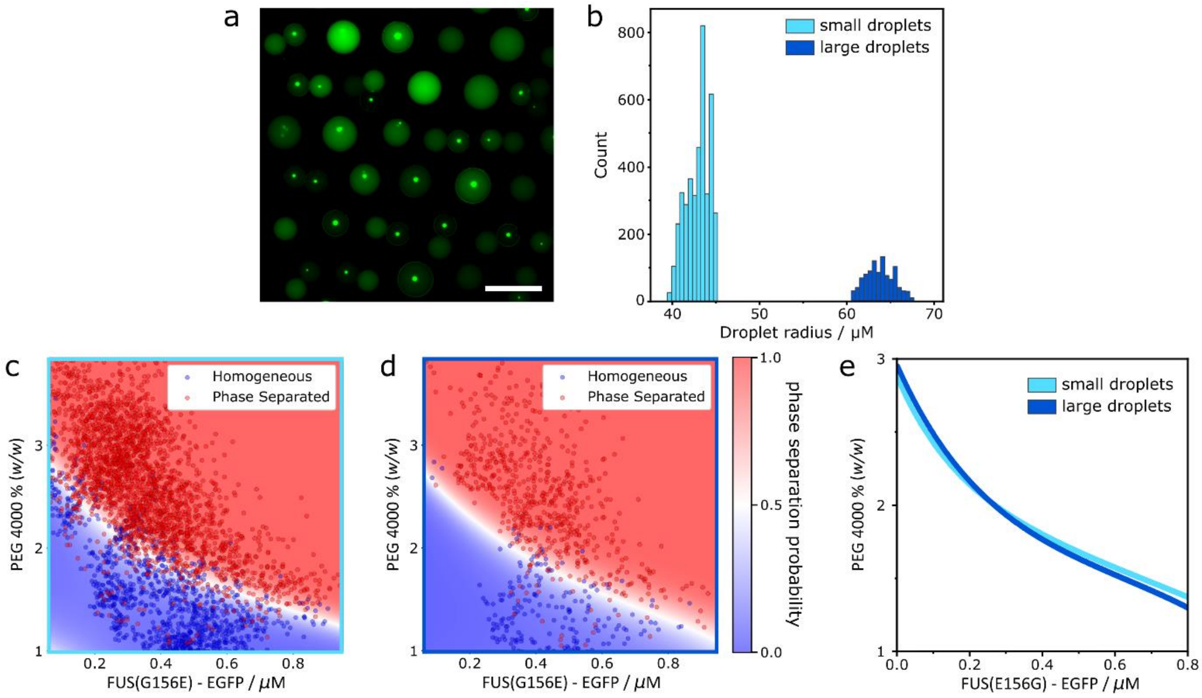

Supplementary Figure 5. Effect of droplet size on condensate phase behaviour. (a) Epifluorescence microscopy images of trapped microdroplets with two different populations of droplet size. Shown is EGFP fluorescence corresponding to EGFP-tagged FUS^{G156E}. **(b)** Histogram of microdroplet size distribution showing a small (*r* = 43 μm) and a large (*r* = 65 μm) droplet population. **(c, d)** Phase diagram of EGFP-tagged FUS^{G156E} vs. PEG concentration as obtained from experiments performed in small (panel c) and large (panel d) microdroplets. **(e)** Overlaying the computed phase boundaries obtained from small and large droplets showed negligible difference in phase behaviour between the two populations. Source data is provided as a Source Data file.

Supplementary Note 5

To investigate whether droplet size influences the observed phase behaviour, an experiment was performed in which a phase diagram for EGFP-FUS^{G156E} and PEG was recorded using two different populations of droplet size (Supplementary Figure 5(a)). Droplets with mean radius of 43 μm (small droplets) and 65 μm (large droplets) were produced by operating the droplet generator with oil flow rates of 300 μL/h and 60 μL/h, respectively (Supplementary Figure 4(b)). Notably, this range of droplet size (3.5-fold difference in volume and 2.3-fold difference in surface area) is much larger than that produced during a conventional PhaseScan experiment, where droplet radii typically vary by <10%. Droplet imaging and analysis was executed as described in the Methods, with the data segregated between the two droplet sizes to afford phase diagrams for each of these droplet populations (Supplementary Figure 5(c, d)). By overlaying the computed phase boundaries for the small and large droplets, it is apparent that no significant difference in phase behaviour between the two populations is observable (Supplementary Figure 5(e)). Phase separation in the binodal region of phase-space occurs through a nucleation/growth mechanism, and phase separation could therefore be assumed to display volume and/or surface dependence as observed previously.[1] However, in the experimental procedure outlined here, droplets are assayed several minutes after generation and mixing. Therefore, we propose that each droplet microenvironment has sufficient time to reach chemical equilibrium, with the PhaseScan measurement invariant to droplet size over the range investigated here.

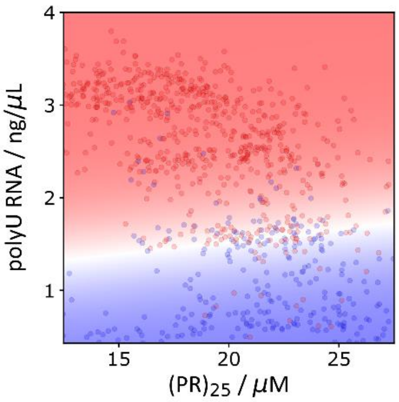

Supplementary Figure 6. PhaseScan characterisation of phase separation of $(PR)_{25}$ and polyU RNA. To demonstrate the applicability of the PhaseScan assay to simple peptide systems as well as full-length proteins, we examined the phase behaviour of a dipeptide repeat system derived from the hexanucleotide repeat expansion in the chromosome 9 open reading frame 72 (C9orf72) gene, implicated in ALS.[2] The peptide consisted of 25 repeats of proline–arginine dipeptide $(PR)_{25}$. This type of peptide is well known to phase separate when mixed with negatively charged polymers, including single-stranded RNA. We assayed the formation of $(PR)_{25}$ with poly uridine ($PolyU_{100}$) RNA and observed RNA-dependent phase separation above 1.5 ng/μL RNA for ~20 μM $(PR)_{25}$. Source data are provided as a Source Data file.

Additional microdroplet generator designs

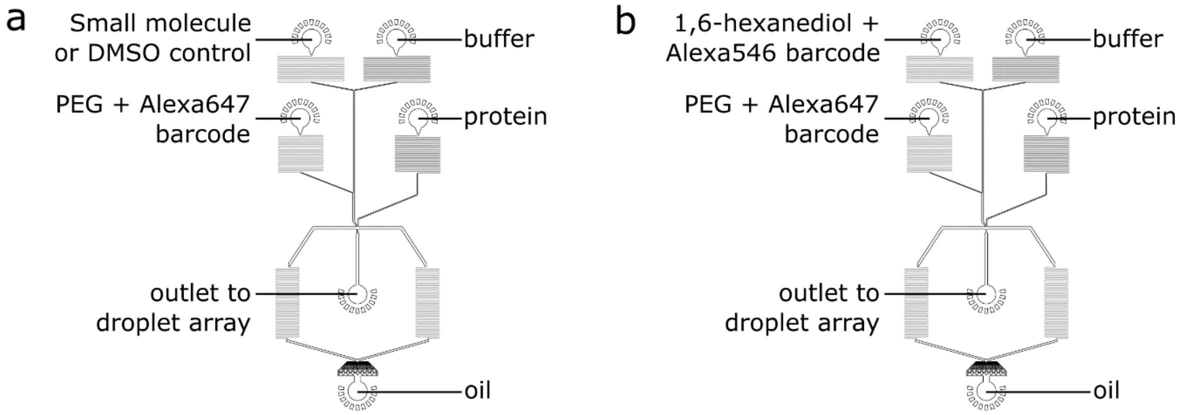

Supplementary Figure 7. Schematics of microfluidic devices for generation of microdroplets containing four aqueous components. (a) Device design and input configuration for analysis of the effect of small molecules on phase separation (Figure 4, main text). **(b)** Device design and input configuration for generation of three-dimensional phase diagrams (Figure 4, main text).

Combined PhaseScan and droplet-shrinking methodologies

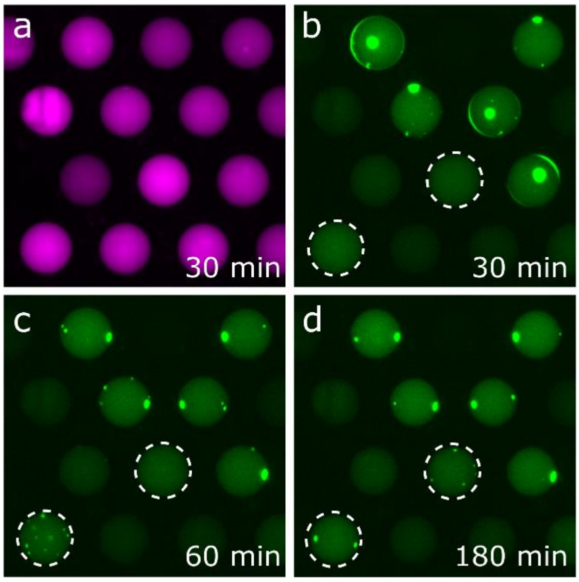

Supplementary Figure 8. Shrinkage of droplets following PhaseScan experiment. (a) Epifluorescence microscopy image of Alexa647 dye encoding polyU RNA concentration 30 min after droplet trapping. **(b–d)** Images of FUS^{G156E}-EGFP fluorescence of phase-separated and homogeneous droplets 30, 60 and 180 min after droplet trapping, respectively. Droplets observed to transition from homogeneous to phase-separated regime are highlighted by dashed outline.

Supporting Materials and Methods

Calibration of droplet fluorescence intensity

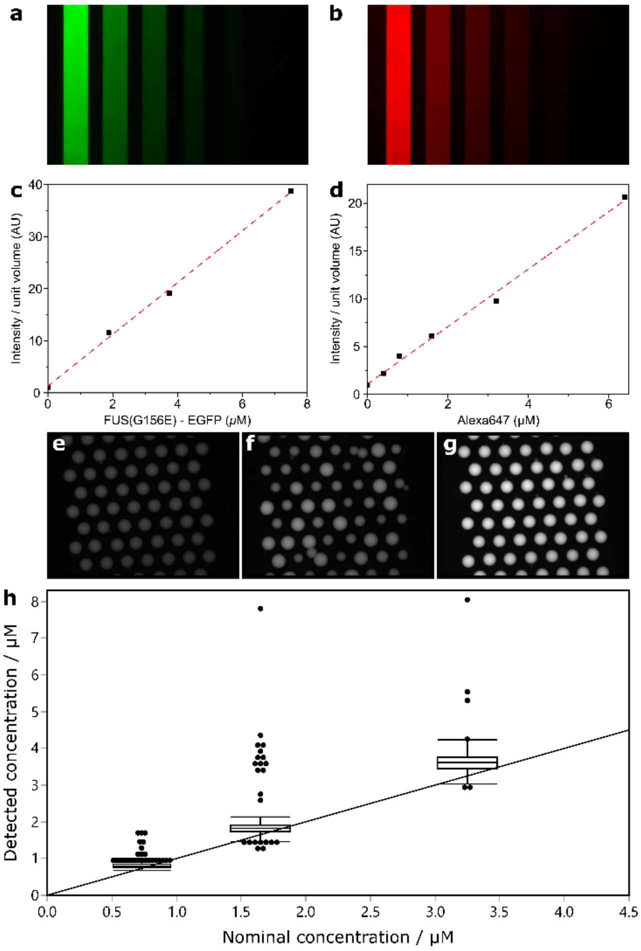

Supplementary Figure 9. Calibration of droplet fluorescence intensity. (a, b) Representative epifluorescence microscopy images of known concentrations of EGFP-tagged FUS^{G156} and Alexa647, respectively, contained in microchannels of known volume. **(c, d)** Calibration of EGFP-tagged FUS^{G156} and Alexa647 concentration and calculated fluorescence intensity per unit volume, respectively. **(e, f, g)** Microdroplets containing Alexa647 at nominal concentrations of 0.73 µM, 1.65 µM and 3.25 µM, respectively. **(h)** Box plot of nominal Alexa647 droplet concentration and concentration as determined by the analysis and intensity–concentration calibration procedure. Box plots were generated from N = 477, 452 and 454 droplets containing nominal Alexa647 concentrations of 0.73 µM, 1.65 µM and 3.25 µM, respectively. Box plots show centre line as median, box limits as upper and lower quartiles, whiskers 95th percentile. Source data are provided as a Source Data file.

Supplementary Note 6

A calibration procedure was employed to convert the intensity per unit volume to the concentration of the corresponding barcode fluorophore (Supplementary Figure 9) in order to allow each droplet to be accurately located in chemical space in the resultant phase diagram. The same fluorophore-containing solutions used in each experiment were injected into microchannels of known dimensions (cross sectional area typically 150 × 30 μm) and were imaged under the same conditions as the droplets (Supplementary Figure 9(a, b)). From these images, the relationship between intensity and fluorophore concentration was determined on an experiment-by-experiment basis (to account for variation in pipetting, imaging, light source intensity etc.), as well as demonstrating the linear relationship between fluorophore concentration and fluorescence intensity (Supplementary Figure 9(c, d)).

To demonstrate the efficacy of this approach, a control experiment was conducted where droplets containing known concentrations of Alexa647 fluorophore (as determined by UV-vis spectroscopy/NanoDrop) were produced, trapped, imaged and subjected to the analysis and calibration procedure (Supplementary Figure 9(e, f, g)). The nominal fluorophore concentration present in each droplet population was observed to be in very good agreement with that determined by the analysis procedure (Supplementary Figure 9(h)).

Detection and classification of droplets and condensates

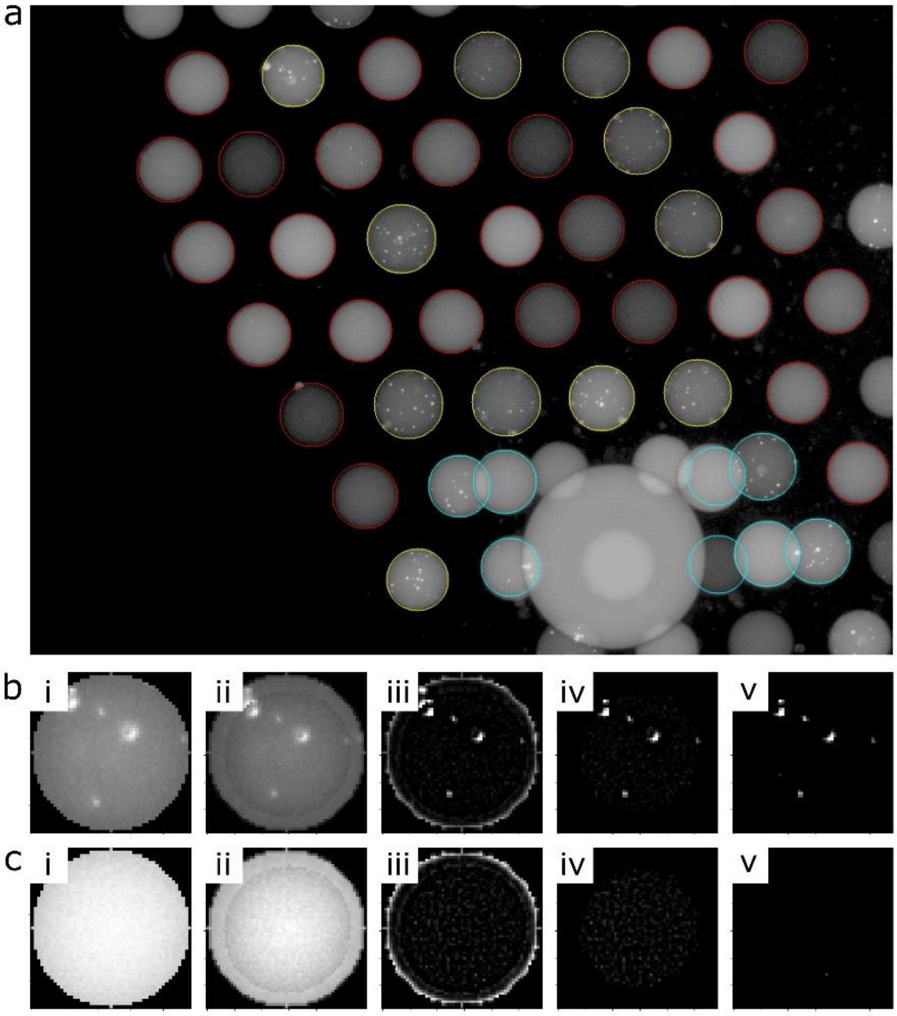

Supplementary Figure 10. Detection and classification of droplets and condensates. (a) Yellow: phase separated droplets, red: homogeneous droplets, cyan: erroneously arrayed droplets. **(b, c)** Example droplet classification procedure for phase separated and homogeneous droplets, respectively. From left to right: (i) detected droplet, (ii) padding, (iii) convolution, (iv) cropping of the padded part, and (v) thresholding and identification of pixel clusters. For any droplet containing a cluster with >1 pixels, the droplet is considered as phase separated, otherwise mixed.

Supplementary Note 7

The collected images were analysed using a custom-written Python script (Supplementary Figure 10). The raw images were processed to remove camera dark noise (Background) and flattened to correct non-uniform epifluorescence illumination by division with a calibration image taken of a homogeneous fluorescent solution (Illumination) according to $Processed\ Image = \frac{Image - Background}{Illumination - Background}$. The raw image was enhanced by taking the logarithm of the

image, applying rolling ball background subtraction, taking the logarithm again, and thresholding the darkest 10% and brightest 10% pixels. Droplets were fitted as circles by finding the bright peaks in the image (Supplementary Figure 10(a)). Any incomplete circles that are partly out of the image boundary as well as those smaller or larger than the radius thresholds were removed. Non-circular droplets or erroneous detections were removed by comparing a corresponding perfect sphere of same size and the image of the droplet, where the brightness multiplied by a proportional constant is used as the z-axis. The total intensity was calculated and normalised to afford intensity per unit volume (calculated using fitted diameter).

To distinguish droplets with condensates formed via phase separation and those which are well-mixed (Supplementary Figure 10(b, c)), the droplet images were convoluted with an edge detection kernel after padding. The result image was made binary with a given threshold ratio of quartiles and medians. If there were at least two connected bright pixels, the droplet was classified as phase separated. Otherwise, the bright pixels were determined as noise and the droplet was labelled as well-mixed. The phase boundary was estimated using a support vector machine (SVM) algorithm with a radial basis function (RBF) kernel. The parameters of SVM were selected according to grid search scores. The parameter space was sampled as a 2D/3D mesh grid and predicted by the SVM model, which was then used to generate iso-boundary or iso-surface as the phase diagram boundary.

Supplementary References

1. Linsenmeier, M. *et al.* Dynamics of Synthetic Membraneless Organelles in Microfluidic Droplets. *Angewandte Chemie International Edition* **58**, 14489–14494 (2019).

2. Van Blitterswijk, M., Dejesus-Hernandez, M. & Rademakers, R. How do C9ORF72 repeat expansions cause amyotrophic lateral sclerosis and frontotemporal dementia: Can we learn from other noncoding repeat expansion disorders? *Current Opinion in Neurology* **25**, 689–700 (2012).

3. Patel, A. *et al.* A Liquid-to-Solid Phase Transition of the ALS Protein FUS Accelerated by Disease Mutation. *Cell* **162**, 1066–1077 (2015).

4. Lemaitre, R. P., Bogdanova, A., Borgonovo, B., Woodruff, J. B. & Drechsel, D. N. FlexiBAC: a versatile, open-source baculovirus vector system for protein expression, secretion, and proteolytic processing. *BMC Biotechnology 2019 19:1* **19**, 1–11 (2019).

5. Guillén-Boixet, J. *et al.* RNA-Induced Conformational Switching and Clustering of G3BP Drive Stress Granule Assembly by Condensation. *Cell* **181**, 346-361.e17 (2020).

6. Borodavka, A., Dykeman, E. C., Schrimpf, W. & Lamb, D. C. Protein-mediated RNA folding governs sequence-specific interactions between rotavirus genome segments. *eLife* **6**, (2017).

7. Bravo, J. P. K. *et al.* Structural basis of Rotavirus RNA Chaperone displacement and RNA annealing. *Proceedings of the National Academy of Sciences of the United States of America* **118**, 1–10 (2021).

8. Geiger, F. *et al.* Liquid–liquid phase separation underpins the formation of replication factories in rotaviruses. *The EMBO Journal* e107711 (2021) doi:10.15252/EMBJ.2021107711.

9. Martin, D., Ouldali, M., Ménétrey, J. & Poncet, D. Structural organisation of the rotavirus nonstructural protein NSP5. *Journal of Molecular Biology* **413**, 209–221 (2011).

10. Wu, F. *et al.* A new coronavirus associated with human respiratory disease in China. *Nature 2020 579:7798* **579**, 265–269 (2020).

11. Mazutis, L. *et al.* Single-Cell Analysis and Sorting Using Droplet-Based Microfluidics. *Nat. Protocols* **8**, 870–891 (2013).

12. Challa, P. K., Kartanas, T., Charmet, J. & Knowles, T. P. J. Microfluidic Devices Fabricated Using Fast Wafer-Scale LED-Lithography Patterning. *Biomicrofluidics* **11**, 014113 (2017).
