# Supplementary material for: Biomolecular condensate phase diagrams with a combinatorial microdroplet platform
Source: Nat Commun. 2022 Dec 21;13:7845. doi: 10.1038/s41467-022-35265-7 (PMC9768726; doi:10.1038/s41467-022-35265-7)
Supplement: Supplementary file 3 — Description of Additional Supplementary Files [file 41467_2022_35265_MOESM3_ESM.pdf]

Title: Supplementary Movie 1
Description: Revolving three-dimensional image of phase diagram of EGFP-tagged FUS^{G156E} vs. PEG 6000 vs. 1,6-HD concentration. N = 3904 droplets.
