# Supplementary material for: Biomolecular condensate phase diagrams with a combinatorial microdroplet platform
Source: Nat Commun. 2022 Dec 21;13:7845. doi: 10.1038/s41467-022-35265-7 (PMC9768726; doi:10.1038/s41467-022-35265-7)
Supplement: Supplementary file 4 — Reporting Summary [file 41467_2022_35265_MOESM4_ESM.pdf]

nature portfolio

Corresponding author(s): Tuomas PJ Knowles

Last updated by author(s): Oct 28, 2022

Reporting Summary

Nature Portfolio wishes to improve the reproducibility of the work that we publish. This form provides structure for consistency and transparency in reporting. For further information on Nature Portfolio policies, see our Editorial Policies and the Editorial Policy Checklist.

Statistics

For all statistical analyses, confirm that the following items are present in the figure legend, table legend, main text, or Methods section.

n/a	Confirmed	
☐	☒	The exact sample size (n) for each experimental group/condition, given as a discrete number and unit of measurement
☐	☒	A statement on whether measurements were taken from distinct samples or whether the same sample was measured repeatedly
☒	☐	The statistical test(s) used AND whether they are one- or two-sided<br>*Only common tests should be described solely by name; describe more complex techniques in the Methods section.*
☒	☐	A description of all covariates tested
☒	☐	A description of any assumptions or corrections, such as tests of normality and adjustment for multiple comparisons
☒	☐	A full description of the statistical parameters including central tendency (e.g. means) or other basic estimates (e.g. regression coefficient) AND variation (e.g. standard deviation) or associated estimates of uncertainty (e.g. confidence intervals)
☒	☐	For null hypothesis testing, the test statistic (e.g. F, t, r) with confidence intervals, effect sizes, degrees of freedom and P value noted<br>*Give P values as exact values whenever suitable.*
☒	☐	For Bayesian analysis, information on the choice of priors and Markov chain Monte Carlo settings
☒	☐	For hierarchical and complex designs, identification of the appropriate level for tests and full reporting of outcomes
☒	☐	Estimates of effect sizes (e.g. Cohen's d, Pearson's r), indicating how they were calculated

Our web collection on statistics for biologists contains articles on many of the points above.

Software and code

Policy information about availability of computer code

Data collection	Metamorph 6.3 image acquisition software
Data analysis	Analysis was conducted with a custom-written Python script (Python version 3.8, SVM algorithms are a component of the scikit-learn Python library) https://github.com/rqi14/PhaseScan

For manuscripts utilizing custom algorithms or software that are central to the research but not yet described in published literature, software must be made available to editors and reviewers. We strongly encourage code deposition in a community repository (e.g. GitHub). See the Nature Portfolio guidelines for submitting code & software for further information.

Data

Policy information about availability of data

All manuscripts must include a data availability statement. This statement should provide the following information, where applicable:

- Accession codes, unique identifiers, or web links for publicly available datasets
- A description of any restrictions on data availability
- For clinical datasets or third party data, please ensure that the statement adheres to our policy

Source data are provided with this paper and are available in the FigShare repository under accession code 10.6084/m9.figshare.21405129. LLPSDB: A Database of Proteins Undergoing Liquid-Liquid Phase Separation in Vitro is available at http://bio-comp.org.cn/llpsdb/home.html

Human research participants

Policy information about studies involving human research participants and Sex and Gender in Research.

Reporting on sex and gender	N/A
Population characteristics	N/A
Recruitment	N/A
Ethics oversight	N/A

Note that full information on the approval of the study protocol must also be provided in the manuscript.

Field-specific reporting

Please select the one below that is the best fit for your research. If you are not sure, read the appropriate sections before making your selection.

[x] Life sciences [ ] Behavioural & social sciences [ ] Ecological, evolutionary & environmental sciences

For a reference copy of the document with all sections, see nature.com/documents/nr-reporting-summary-flat.pdf

Life sciences study design

All studies must disclose on these points even when the disclosure is negative.

Sample size	Since the speed of data generation was a key parameter for the methodology presented in the manuscript, sample size was determined by the number of analyzable data points (see data exclusions) after ~5 min of droplet generation. This resulted in data sets of >1500 data points for each experiment. This was found to be a sufficient sample size since increasing the sample size further did not result in a significant change in the reported phase behaviour (e.g. comparison of Fig. S2 and Fig. S4).
Data exclusions	In image analysis of trapped microdroplets, data were excluded for overlapping or touching droplets since these effects prevent quantitative droplet analysis. Examples of this process are shown in Fig. S10.
Replication	We confirm that experiment replicates were successful. Example replicate experiments are shown in Fig. S2 and Fig. S4
Randomization	Droplet collection took place over a significantly longer timescale than the flow profile required to produce the phase diagram, so that microdroplets generated according to the flow profile were sampled evenly. All trapped microdroplets were imaged, and imaged and analysed equally, with no sub-sampling bias. There was thus no requirement for randomization.
Blinding	Quantification was performed using an automated analysis pipeline applied equally to all conditions and replicates for a given experiment. A subset of the data was analysed in a blinded experiment, which produced the same output as in the non-blinded analysis.

Reporting for specific materials, systems and methods

We require information from authors about some types of materials, experimental systems and methods used in many studies. Here, indicate whether each material, system or method listed is relevant to your study. If you are not sure if a list item applies to your research, read the appropriate section before selecting a response.

Materials & experimental systems

n/a	Involved in the study	
[x]	[ ]	Antibodies
[ ]	[x]	Eukaryotic cell lines
[x]	[ ]	Palaeontology and archaeology
[x]	[ ]	Animals and other organisms
[x]	[ ]	Clinical data
[x]	[ ]	Dual use research of concern

Methods

n/a	Involved in the study	
[x]	[ ]	ChIP-seq
[x]	[ ]	Flow cytometry
[x]	[ ]	MRI-based neuroimaging

Eukaryotic cell lines

Policy information about cell lines and Sex and Gender in Research

Cell line source(s)	sf9 cell line (Expression Systems)
Authentication	Authenticated at source
Mycoplasma contamination	All cell lines tested negative for mycoplasma contamination
Commonly misidentified lines (See ICLAC register)	None
